# Theoretical Study of Single-Atom Catalysts for Hydrogen Evolution Reaction Based on BiTeBr Monolayer

**DOI:** 10.3390/ma17102377

**Published:** 2024-05-15

**Authors:** Tao Yang, Qiquan Luo

**Affiliations:** Institutes of Physical Science and Information Technology, Anhui University, Hefei 230601, China; qluo@ustc.edu.cn

**Keywords:** density functional theory, hydrogen evolution reaction, single-atom catalysts, constant potential

## Abstract

Developing an inexpensive and efficient catalyst for a hydrogen evolution reaction (HER) is an effective measure to alleviate the energy crisis. Single-atom catalysts (SACs) based on Janus materials demonstrated promising prospects for the HER. Herein, density functional theory calculations were conducted to systematically investigate the performance of SACs based on the BiTeBr monolayer. Among the one hundred and forty models that were constructed, fourteen SACs with potential HER activity were selected. Significantly, the SAC, in which a single Ru atom is anchored on a BiTeBr monolayer with a Bi vacancy (Ru_S2_/V_Bi_-BiTeBr), exhibits excellent HER activity with an ultra-low |Δ*G*_H*_| value. A further investigation revealed that Ru_S2_/V_Bi_-BiTeBr tends to react through the Volmer–Heyrovsky mechanism. An electronic structure analysis provided deeper insights into this phenomenon. This is because the Tafel pathway requires overcoming steric hindrance and disrupting stable electron filling states, making it challenging to proceed. This study finally employed constant potential calculations, which approximate experimental situations. The results indicated that the Δ*G*_H*_ value at pH = 0 is 0.056 eV for Ru_S2_/V_Bi_-BiTeBr, validating the rationality of the traditional Computational Hydrogen Electrode (CHE) method for performance evaluation in this system. This work provides a reference for the research of new HER catalysts.

## 1. Introduction

Currently, the global demand for energy is experiencing rapid expansion due to population growth and industrial development. However, traditional fossil fuels, with their finite supply and detrimental environmental impact, are no longer sustainable options. Consequently, there is a pressing need to explore and develop renewable and clean energy sources to mitigate the looming energy crises. This urgency has ignited significant scientific endeavors aimed at advancing sustainable alternative energy technologies to meet the escalating energy demands of society while reducing greenhouse gas emissions and environmental degradation. Hydrogen, as a new energy carrier, has become the focal point of widespread interest due to its exceptional energy density and eco-friendly attributes [1,2]. The utilization of intermittent energy sources such as wind and tidal power to produce hydrogen via water electrolysis represents an advanced technology in the realm of renewable energy conversion. This innovative approach not only addresses the intermittency issues associated with renewable energy but also underscores the potential of hydrogen as a versatile and sustainable energy vector for various applications [3,4]. The hydrogen evolution reaction (HER) serves as the cathodic half-reaction of electrochemical water splitting. Traditionally, platinum (Pt) is the primary electrocatalyst for the HER [5,6]. Yet, Pt’s scarcity and high cost make it impractical for large-scale manufacturing. As a result, researchers have been actively seeking novel catalysts that are readily available and cost-effective as alternatives to Pt [7,8].

Single-atom catalysts (SACs) have been successfully applied for the catalysis of various reactions due to their high activity and selectivity [9,10,11,12,13,14], opening up new avenues for the design of cost-effective and highly efficient electrocatalysts. Recently, sandwiched monolayer Janus materials have been successfully experimentally fabricated by utilizing the improved chemical vapor deposition (CVD) method. During the selenization process, the S atoms on the top layer for MoS_2_ are substituted with Se atoms, resulting in the creation of a Janus MoSSe monolayer with good structural stability [15,16]. Janus materials show distinct atom arrangements on each side, breaking mirror symmetry in relation to the basal plane. Janus materials generate a built-in electric field due to the unique mirror asymmetry, which may give these new materials unique properties, such as a large piezoelectric response, strong Rashba spin splitting, and good catalytic performance [16,17,18,19,20,21]. Lu’s research team recently discovered that catalysts, incorporating As and Si atoms into the S or Se sites of VSSe, as well as C and Ge atoms into the Se site of VSSe, demonstrate exceptional performance in the HER [22]. Wang et al. performed theoretical calculations to explore the anchoring of transition metal (TM) atoms on the Janus MoSSe surface, with the aim of identifying effective SACs for the HER [23]. Their research noted that anchoring a single Fe atom on the Janus MoSSe surface exhibited exceptional performance in the HER. Xiao et al. reported that a single Zn atom anchored on the Janus MoSSe monolayer containing a S vacancy exhibits prominent HER activity [24]. These studies have all shown the feasibility of SACs based on Janus materials for electrocatalytic hydrogen evolution and exhibit excellent HER activity. Two-dimensional BiTeBr is an intrinsic Janus semiconductor with many exotic surface physical properties [25,26,27,28]; the SACs based on the BiTeBr monolayer are expected to have excellent HER prospects.

Understanding the HER mechanisms is crucial for advancing both the fundamental science and practical applications in electrocatalysis. The HER can potentially proceed through two distinct mechanistic routes: the Volmer–Heyrovsky mechanism and Volmer–Tafel mechanism. The initial step in both routes involves the electrochemical adsorption of hydrogen atoms onto the catalyst surface, which is described by the Volmer reaction as H^+^ + e^−^ = H*. Once hydrogen atoms are adsorbed, they can further react to form hydrogen gas via two different pathways. The first pathway involves the Heyrovsky reaction, which is an electrochemical desorption process. In this mechanism, an adsorbed hydrogen atom combines with a proton and an electron to form a hydrogen molecule, represented as H* + H^+^ + e^−^ = H_2_. The second pathway is known as the Tafel reaction, which is a chemical desorption process. In this route, two adsorbed hydrogen atoms come together to form H_2_ (H* + H* = H_2_) [29,30]. Elucidating these mechanisms offers insights into electrode surface processes and is pivotal for designing and optimizing effective catalysts.

In this work, the SACs were first constructed by anchoring a series of TM single atoms (from V to Cu, from Nb to Ag, and from Ta to Au, excluding the radioactive Tc) on the BiTeBr monolayer containing a vacancy as well as a perfect BiTeBr monolayer. The stability and HER activity of the constructed SACs were assessed via theoretical calculations, resulting in the identification of fourteen potential HER catalysts from a pool of one hundred and forty configurations. Among them, the structure in which a single Ru atom is anchored on a BiTeBr monolayer with a Bi vacancy (Ru_S2_/V_Bi_-BiTeBr) shows the best HER activity. Additionally, the specific mechanism of the HER for Ru_S2_/V_Bi_-BiTeBr was explored. The results show that Ru_S2_/V_Bi_-BiTeBr is more conducive to the reaction through the Volmer–Heyrovsky mechanism rather than the Volmer–Tafel mechanism. The electronic structure analysis elucidates the reason behind the facile nature of this mechanism. This is attributed to the need to overcome steric hindrance and disrupt stable electronic filling states, as demanded by the Tafel mechanism. In contrast to the traditional calculation assuming constant charge conditions, the constant potential method was finally employed to simulate the situation closer to the experimental reaction conditions, and the results indicate that the Δ*G*_H*_ value at pH = 0 is 0.056 eV for Ru_S2_/V_Bi_-BiTeBr. Meanwhile, the reasonableness of the traditional Computational Hydrogen Electrode (CHE) method for performance evaluation in this system is confirmed.

## 2. Materials and Methods

All the spin-polarized density functional theory (DFT) calculations were performed by using the Vienna ab initio simulation package (VASP5.4.4) [31]. The projector augmented-wave (PAW) pseudopotential method was adopted [32,33]. The exchange–correlation interaction was described using the Perdew−Burke−Ernzerhof (PBE) functional within the generalized gradient approximation (GGA). A plane wave basis with an energy cutoff of 450 eV was used. In two-dimensional materials, Van der Waals (vdW) forces play a significant role in both the structure and properties. Therefore, when performing calculations on two-dimensional materials, the influence of Van der Waals forces must be taken into account. Grimme’s DFT-D3 approach was used to modify the vdW dispersion interactions [34,35]. A 4 × 4 supercell was chosen, and the vacuum layer of 15 Å was added in the z direction to avoid periodic interactions. The geometry optimization was terminated when the forces on the atoms were smaller than 0.02 eV/Å and the total energy convergence reached less than 1 × 10^−5^ eV. Furthermore, 2 × 2 × 1 and 4 × 4 × 1 gamma-centered k-points mesh were used to represent the Brillouin zone for geometric optimizations and electronic structure calculations, respectively. After generating vacancies, we allowed the system to undergo adequate relaxation to achieve a more stable configuration. The relaxation process was performed using the same convergence criteria as used for the structural optimization to ensure consistency. The phonon dispersion was computed to assess the dynamic stability of the catalyst by using density functional perturbation theory (DFPT) [36] and the Γ, M, and K serving as the special points in the reciprocal space for the calculations. The HSE06 hybrid functional method was used to calculate the density of the states for the catalysts based on the BiTeBr monolayer. The nudged elastic band (NEB) method was used for the transition state searching.

To measure the possibility of forming the single Te/Bi/Br vacancy on the BiTeBr monolayer, the formation energy (*E*_f_) can be defined as:

(1) $$E_{f}=E_{V}-E_{\mathrm{perfect}}+\mu_{\mathrm{Te}/\mathrm{Bi}/\mathrm{Br}},$$

where *E*_V_ and *E*_perfect_ are the total energy of V_Te_-BiTeBr/V_Bi_-BiTeBr/V_Br_-BiTeBr and the perfect slab, respectively, and *μ*_Te/Bi/Br_ is the chemical potential of the Te/Bi/Br atom.

The binding energy (*E*_b_) was used to evaluate the structural stability of the SACs, which is defined as:

(2) $$E_{b}=E_{\mathrm{SAC}}-E_{\mathrm{slab}}-E_{\mathrm{TM}},$$

Here, *E*_SAC_, *E*_slab_, and *E*_TM_ are the total energy of the SACs, slab, and single TM atom, respectively. More negative binding energy corresponds to more stable structures.

To evaluate the HER activity of the SACs, the adsorption free energy of H (Δ*G*_H*_) was calculated at equilibrium using:

(3) $$\Delta G_{H*}=\Delta E+\Delta ZPE-T\Delta S,$$

Here, Δ*E*, Δ*ZPE*, and *T*∆*S* are the hydrogen adsorption energy, the zero-point energy difference between the adsorbed hydrogen and molecular hydrogen in the gas phase, and the entropy difference between adsorbed H and H_2_ in the gas phase, respectively. For computational convenience, we converted Δ*E* into the Δ*G*_H*_ scale using the previously proposed relation as [37]:

(4) $$\Delta G_{H*}=\Delta E+0.24,$$

The theoretical exchange current *i*_0_ was calculated under the equilibrium potential *U* = 0 and pH = 0 conditions by:

(5) $$i_{0}=-ek_{0}/\left[1+\exp(\left|\Delta G_{H*}\right|/k_{b}T)\right],$$

Here, *k*_0_ is the reaction rate constant at zero overpotential, *k*_b_ is the Boltzmann constant, and *T* is the temperature. For illustrative purposes, *k*_0_ was set to 1.

For the constant potential calculation of Ru_S2_/V_Bi_-BiTeBr, the solvent environment was simulated using the VASPsol code with a relative permittivity of 78.4 to simulate the aqueous electrolyte [38]. To explore the reactions at varying electrode potentials, we adjusted the excess charge of the cell from −0.5 e to +0.5 e in increments of 0.1 e. The specific calculation steps of the constant potential method can be found in the Supplementary Materials.

## 3. Results and Discussion

### 3.1. Structural Model and Hydrogen Adsorption Ability of Janus BiTeBr Monolayer

**Figure 1:**
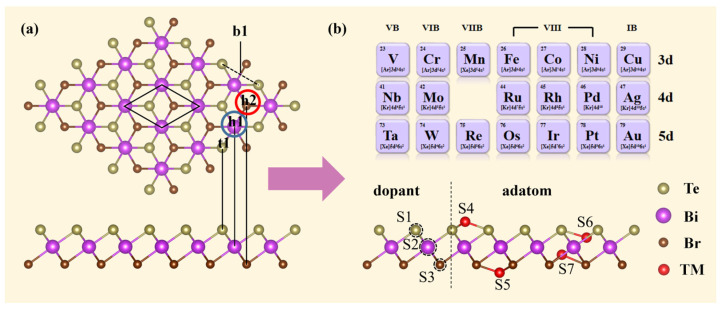
(**a**) The top and side views of a 4 × 4 supercell of the Janus BiTeBr monolayer structure and possible hydrogenation adsorption sites on the Te-terminated side: t1, b1, h1, and h2; likewise, there are four counterpart sites on the Br-terminated side. The unit cell is represented by a solid black rhombus line. These eight adsorption sites all have the Δ*G*_H*_ > 1.6 eV, indicating that the pristine monolayer is inactive in the HER. (**b**) Selected TM for constructing SACs and their possible locating sites on the monolayer. S1~S7 represent the 3 doped sites and 4 adatom sites, respectively. Te atoms in gray, Bi atoms in pink, Br atoms in brown, and selected TM atoms in red, respectively.

The geometrical structure of the Janus BiTeBr monolayer is shown in Figure 1a, where a Bi atom is situated between a Te atom and a Br atom within the unit cell, forming a sandwich-like configuration. The space group of the BiTeBr unit cell is P3m1, and the initial coordinates for the DFT calculation are provided in the Supplementary Materials [39]. The optimized lattice parameters (hexagonal) are a = b = 4.33 Å; the Te-Bi and Br-Bi bond lengths are 3.04 Å and 3.12 Å, respectively, and the computed results are consistent with previous reports [35,40]. The hydrogen adsorption ability to cathode could be regarded as a primary assessment of the HER activity, according to the profound CHE model [37]. A total of eight possible hydrogen adsorption sites were considered. On the Te-terminated side, the top site t1 of the Te atom, the bridge site b1 of the two Te atoms, and two different hollow sites h1 (hcp) and h2 (fcc) were considered, as depicted in Figure 1a. The sites on the Br-terminated side are similarly noted as t2, b2, h3, and h4, respectively. The Δ*G*_H*_ values on these considered sites all exceed 1.6 eV (Table 1), indicating the pristine Janus BiTeBr monolayer is inert to the reaction. Consequently, TM_1_ doping and as adatoms were chosen to modify the Janus BiTeBr monolayer structure, creating SACs aimed at enhancing catalytic activity.

### 3.2. The Stability of SACs Based on BiTeBr Monolayer

Two-dimensional materials are used as supports to construct SACs, enabling experimental access to both doping methodologies and the use of single atoms as adatoms. This study initially explored three potential vacancies (S1~S3) in the BiTeBr monolayer, identified as V_Te_-BiTeBr, V_Bi_-BiTeBr, and V_Br_-BiTeBr, illustrated in Figure 1b. The calculated *E*_f_ for these vacancies is 3.58 eV, 1.78 eV, and 2.27 eV, respectively. For comparison, the *E*_f_ of a single carbon vacancy in graphene was estimated at 7.5 eV [41,42]. Consequently, SACs synthesized on the BiTeBr monolayer featuring any of these three types of vacancies are anticipated to form under specific preparation conditions. TM_1_ anchored to the vacant site of V_Te_-BiTeBr, V_Bi_-BiTeBr, and V_Br_-BiTeBr was investigated, denoted as TM_S1_/V_Te_-BiTeBr, TM_S2_/V_Bi_-BiTeBr, and TM_S3_/V_Br_-BiTeBr, respectively, and is depicted in Figure 1b. Regarding TM_1_ as an adatom on the monolayer, four potential adsorption sites were identified. For the four potential adsorption sites (S4~S7) for the TM atom, the S4 site is the hcp site on the Te-terminated side; the S5 site is the hcp site on the Br-terminated side; the S6 site is the fcc site on the Te-terminated side; and the S7 site is the fcc site on the Br-terminated side. The consequent forming SACs are labeled as TM_S4_/BiTeBr, TM_S5_/BiTeBr, TM_S6_/BiTeBr, and TM_S7_/BiTeBr, also shown in Figure 1b.

**Figure 2:**
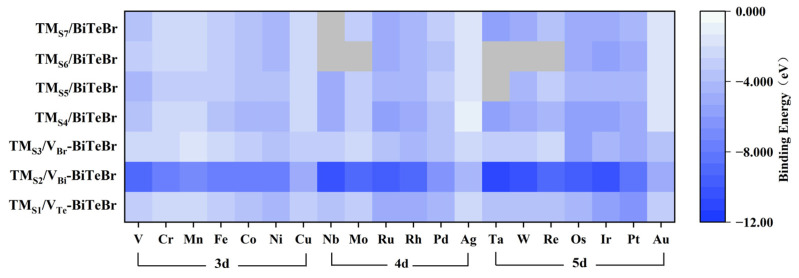
The binding energy for the constructed SACs. The darker areas in the figure indicate stronger binding strength. The grey areas show that the corresponding values were not available due to the significant geometric distortion during the calculations.

The stability of these SACs was initially assessed by calculating the single atom’s binding energy in which less than 0 eV is considered stable [43,44]. The binding energy values range from −11.26 to −1.21 eV, with the results summarized in Figure 2, indicating good stability for SACs. With an increase in the number of *d* orbital electrons in the dopant/adatom within the same period, the binding strength initially decreases, reaching a minimum at half-filled *d* orbitals. As the *d* electrons increase further, the binding strength stabilizes, and the elements with fully filled *d* orbitals demonstrate relatively weaker adsorption. This finding suggests that TM_1_ in stable half-filled and fully filled electronic states may exhibit less stable binding with the substrate [45]. Among these models, TM_S2_/V_Bi_-BiTeBr shows a more stable trend, thanks to the hexacoordinated structure at its dopant sites. For SACs with a single-atom adatom on the Te-termination, TM_S4_/BiTeBr is slightly more stable than TM_S6_/BiTeBr; on the Br-termination, TM_S5_/BiTeBr and TM_S7_/BiTeBr have very similar energy levels. Furthermore, significant geometric distortion in some TM_S6_/BiTeBr SACs means their binding energies could not be obtained for inclusion in Figure 2.

### 3.3. Catalytic Activity of SACs

**Figure 3:**
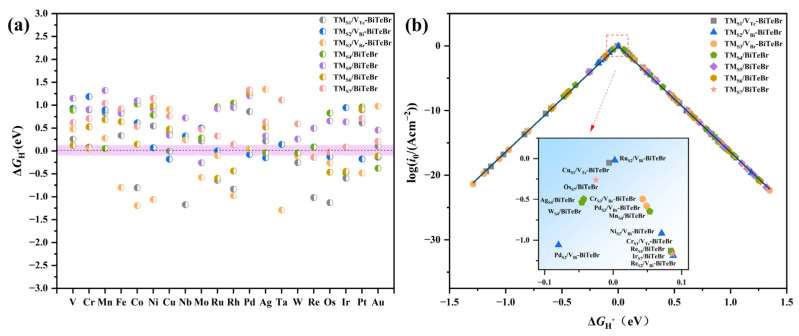
(**a**) The calculated adsorption free energy for different models. The pink area indicates |Δ*G*_H*_| < 0.10 eV. (**b**) The volcano curve of the exchange current i0 as a function of Δ*G*_H*_ for the HER. The inset in (**b**) displays a local zoom-in around the peak of the volcano curve. In total, fourteen SACs were screened out as potential HER catalysts.

Following an investigation into the stability of SACs, their catalytic activity in the HER was primarily explored. The Δ*G*_H*_ serves as a metric for assessing the catalytic activity of an electrocatalyst, with an ideal catalyst exhibiting a Δ*G*_H*_ value that approximates zero. The calculated Δ*G*_H*_ values are presented in Figure 3a. Simultaneously, to visually depict and quantitatively evaluate the catalytic activity of SACs toward the HER, a volcano curve was generated by plotting *i*_0_ as a function of Δ*G*_H*_ (Figure 3b) [46]. The Δ*G*_H*_ values are in the range of −1.29 to 1.35 eV. Compared to the Δ*G*_H*_ values for the pristine BiTeBr monolayer, it is indicated that anchoring single TM atoms activates the material’s basal plane. The HER activity of the catalyst improves as Δ*G*_H*_ approaches the volcano peak; the peak of the volcano curve represents the preferred catalytic region [47]. In this work, a strict criterion |Δ*G*_H*_| < 0.10 eV was used to identify the optimal catalysts. As shown in Figure 3a, the Δ*G*_H*_ values of Pd_S2_/V_Bi_-BiTeBr, W_S4_/BiTeBr, Ag_S4_/BiTeBr, Os_S7_/BiTeBr, Cu_S1_/V_Te_-BiTeBr, Ru_S2_/V_Bi_-BiTeBr, Cr_S3_/V_Br_-BiTeBr, Pd_S3_/V_Br_-BiTeBr, Mn_S4_/BiTeBr, Ni_S2_/V_Bi_-BiTeBr, Cr_S1_/V_Te_-BiTeBr, Re_S4_-BiTeBr, Ir_S7_/BiTeBr, and Re_S2_/V_Bi_-BiTeBr satisfy the condition; thus, their positions are around the peak of the volcano curve (Figure 3b). Fourteen SACs were screened out with the potential to be good cathode materials. Obviously, Ru_S2_/V_Bi_-BiTeBr exhibiting the maximum exchange current was selected for further investigation in the subsequent sections.

### 3.4. Reaction Mechanism for Ru_S2_/V_Bi_-BiTeBr

**Figure 4:**
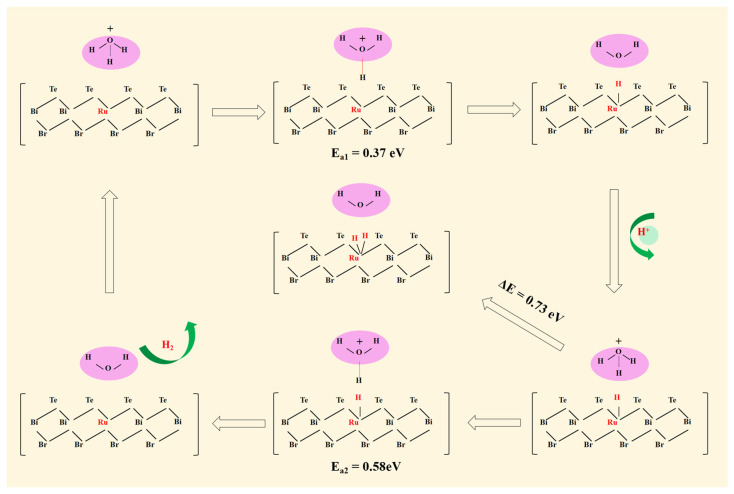
The schematic diagram of the HER for Ru_S2_/V_Bi_-BiTeBr as well as the corresponding activation barrier (*E*_a_) and adsorption energy.

To investigate the proton transfer process during the HER and determine the corresponding mechanism for Ru_S2_/V_Bi_-BiTeBr, the explicit water molecule models were utilized. The explicit water molecule models have been successfully applied in the prior literature [48,49,50]. It is worth mentioning that in the explicit water molecule models with a few water molecules, variations in the configuration, orientation, and number of water molecules can have a significant impact on the system’s energy [51]. Thus, the several common explicit water molecule models of the system with H_3_O^+^ + (H_2_O)_n_ for n = (0, 4, 5) were all constructed. Comparing the reaction energy between the CHE models and the corresponding explicit models (see Table 2), it was found that the reaction energy (−0.51 eV) of the system with H_3_O^+^ is close to the CHE model (−0.24 eV) [52]. For the other two explicit water molecule models, the reaction energy difference between their explicit models and the CHE model is relatively large. Consequently, the explicit model containing H_3_O^+^ was considered more suitable for this system and was chosen. The process of the HER on the Ru_S2_/V_Bi_-BiTeBr surface is illustrated in Figure 4. The H_3_O^+^ from the electrolyte first reacts with an electron, forming an adsorbed H at the Ru single-atom site. The activation energy for the Volmer reaction was calculated with a low barrier of 0.37 eV, indicating that this step is easily achievable. For the Tafel pathway, it subsequently requires the adsorption of another H atom at the Ru single-atom site. However, the adsorption energy is higher for 0.73 eV, indicating that the Tafel step is thermodynamically unfavorable. For the Heyrovsky pathway, H* reacts with a new pair of H_3_O^+^ + e^−^ to produce H_2_. The activation barrier required for the Heyrovsky reaction has been calculated through a transition state search and it is 0.58 eV, which is easy to overcome. The results demonstrate that Ru_S2_/V_Bi_-BiTeBr is more conducive to the reaction through the Volmer–Heyrovsky mechanism.

### 3.5. Analysis of Electronic Structures

**Figure 5:**
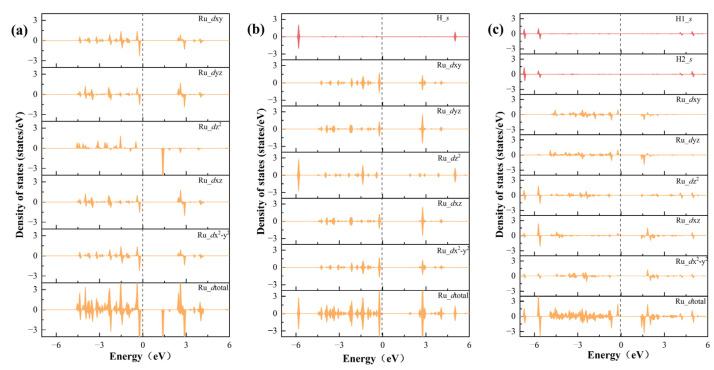
The PDOS of the *d* orbitals of Ru and the *s* orbitals of H in Ru_S2_/V_Bi_-BiTeBr (**a**), Ru_S2_/V_Bi_-BiTeBr*H (**b**), and Ru_S2_/V_Bi_-BiTeBr*2H (**c**).

Electronic structure calculations were performed to elucidate the hydrogen adsorption behavior on the Ru_S2_/V_Bi_-BiTeBr surface and explain the inherent reasons for the HER to easily proceed through the Volmer–Heyrovsky mechanism. The projected density of states (PDOSs) indicates that the Ru atom in Ru_S2_/V_Bi_-BiTeBr contains five valence electrons (4d^5^). At the same time, combining with the outermost electron structure of Ru (4d^7^5s^1^), we infer that Ru exhibits a +3 oxidation state. Owing to the inherent built-in electric field of BiTeBr, the *d* orbital of the Ru atom experiences energy-level splitting. The *d*xz and *d*yz orbitals, sharing an electron pair, are degenerate, a condition that also pertains to the *d*xy and *d*x^2^-y^2^ orbitals, while a single electron occupied the *d*z^2^ orbital, resulting in Ru exhibiting a magnetic moment of 1 μB, as illustrated in Figure 5a. When a H atom is adsorbed, the *s* orbital of the H atom is well hybridized with the *d*z^2^ orbital of Ru (Figure 5b). The interaction between these two orbitals forms an electron pair in the *d*z^2^ orbital and thus reduces Ru’s magnetic moment to 0 μB.

If Ru_S2_/V_Bi_-BiTeBr was to undergo hydrogen evolution through the Tafel mechanism, it would require the re-adsorption of an additional H atom at the Ru single-atom site. Upon the adsorption of the second H atom, an extra electron is introduced into the *d* orbital of the Ru atom, leading to the splitting of the *d* orbital degeneracy and disrupting the previously stable electronic filling state (Figure 5c). This results in the presence of an unpaired electron in the *d* orbital, and the magnetic moment becomes 1 μB. In addition, due to steric hindrance, the presence of the H atom at the Ru site obstructs the adsorption of the second H, rendering the adsorption of the second H an unstable process, making it difficult for Ru_S2_/V_Bi_-BiTeBr to evolve hydrogen through the Tafel mechanism.

### 3.6. Constant Potential

**Figure 6:**
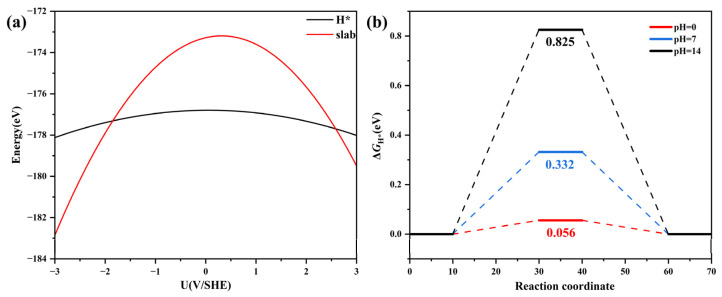
(**a**) The calculated energy of the Ru_S2_/V_Bi_-BiTeBr slab (red) and the corresponding hydrogen adsorption structures (H*, black) as a function of the applied electrode potential. (**b**) A free energy diagram of the HER catalyzed by Ru_S2_/V_Bi_-BiTeBr at different pH.

New challenges arise in exploring chemical reactions that involve the transfer of charge, such as the Volmer reaction and Heyrovsky reaction. In a real system, at the atomic scale, the interface area can be considered infinitely large, thereby keeping the electrode potential fixed in a single charge transfer reaction [53]. However, in the traditional CHE model, all the calculations were conducted under the condition of constant charge. The variations in potential occur during the charge transfer reactions due to the relatively small, simulated structure used in the calculation, which may have an impact on the calculation results. In this work, the double-reference method was employed to investigate the HER over the Ru_S2_/V_Bi_-BiTeBr surface under constant potential conditions [54]. By varying the electron quantity in the system, we simulated the electrochemical double layer as a function of potential in the implicit solvent. At the same time, the relationship between the catalytic activity and pH was elucidated. The calculated energies, plotted against the electrode potential (referenced to the Standard Hydrogen Electrode scale), are presented in Figure 6a, and the energy-potential points exhibit a good quadratic fit. The energy variation with potential is minimal for H* compared to the slab. This indicates that the energy of the slab has a significant response to the electric potential. The relationship between the catalytic activity and pH is illustrated in Figure 6b, the Δ*G*_H*_ values increase with rising *pH,* and the Δ*G*_H*_ at pH = 0 is determined to be 0.056 eV < 0.10 eV. The phonon dispersion was further calculated and is depicted in Figure S1, affirming the dynamic stability of Ru_S2_/V_Bi_-BiTeBr with no imaginary frequencies. Generally, Ru is less expensive than Pt. Simultaneously, single-atom catalysts enhance atom utilization, leading to cost savings compared to bulk Pt catalysts and reduced Ru consumption. The results suggest that Ru_S2_/V_Bi_-BiTeBr is a highly promising cathodic material, validating the rationality of the traditional CHE method for performance evaluation.

## 4. Conclusions

In this work, to enhance the HER catalytic activity, the SACs were constructed by anchoring a series of TM single atoms (from V to Cu, from Nb to Ag, and from Ta to Au, excluding the radioactive Tc), both on the BiTeBr monolayer containing a vacancy and the perfect BiTeBr monolayer. The specific findings are summarized as follows: (1) DFT calculations were employed to conduct high-throughput screening for identifying excellent SACs based on the BiTeBr monolayer for the HER. Fourteen highly active catalysts were screened from a pool of one hundred and forty configurations using the strict criterion |Δ*G*_H*_| < 0.10 eV. (2) Among these fourteen catalysts, the optimal catalyst, Ru_S2_/V_Bi_-BiTeBr, was selected for an in-depth investigation into its reaction mechanism. This study found that Ru_S2_/V_Bi_-BiTeBr exhibits a favorable pathway via the Volmer–Heyrovsky mechanism rather than the Volmer–Tafel mechanism. (3) The results of the electronic structure analysis indicate that Ru_S2_/V_Bi_-BiTeBr is difficult to undergo an HER via the Volmer–Tafel mechanism attributed to steric hindrance and the necessity to disrupt stable electronic filling states. (4) The constant potential method reveals that under actual reaction conditions, Ru_S2_/V_Bi_-BiTeBr still exhibits excellent HER activity, confirming the reasonableness of the conventional CHE model for assessing the HER performance of Ru_S2_/V_Bi_-BiTeBr. On the whole, Ru_S2_/V_Bi_-BiTeBr exhibits excellent HER electrocatalytic activity and is a very promising material. This study offers insights into exploring novel catalysts for the HER.

## Figures and Tables

**Table 1:** The adsorption free energy values for eight possible hydrogenation adsorption sites on the BiTeBr monolayer.

Sites	t1	b1	h1	h2	t2	b2	h3	h4
Δ*G*_H*_ (eV)	1.97	1.77	2.12	1.67	1.76	1.86	1.86	2.32

**Table 2:** The reaction energy of the CHE model and explicit water molecule models of the system with H_3_O^+^ + (H_2_O)_n_ for n = (0, 4, 5) and the unit of reaction energy is eV.

Models		Δ*E*
CHE model		−0.24
Explicit models	H_3_O^+^	−0.51
	H_3_O^+^ + (H_2_O)_4_	0.34
	H_3_O^+^ + (H_2_O)_5_	0.89

## Data Availability

Data are contained within the article and Supplementary Materials.

## Supplementary Materials

The following supporting information can be downloaded at: https://www.mdpi.com/article/10.3390/ma17102377/s1. Figure S1: The phonon dispersion curves for Ru_S2_/V_Bi_-BiTeBr; Table S1: The Δ*G*_H*_ values of different DFT calculation methods for Ru_S2_/V_Bi_-BiTeBr. References [20,35,55,56,57,58] are cited in the Supplementary Materials.
