# Supplementary material for: Theoretical Study of Single-Atom Catalysts for Hydrogen Evolution Reaction Based on BiTeBr Monolayer
Source: Materials (Basel). 2024 May 15;17(10):2377. doi: 10.3390/ma17102377 (PMC11123116; doi:10.3390/ma17102377)
Supplement: Supplementary file 1 [file materials-17-02377-s001.zip › materials-2947576-supplementary.pdf]

Supplementary Materials

Theoretical Study of Single-Atom Catalysts for Hydrogen Evolution Reaction Based on BiTeBr Monolayer

Tao Yang * and Qiquan Luo

Institutes of Physical Science and Information Technology, Anhui University, Hefei 230601, China; qluo@ustc.edu.cn
*Correspondence: y18856420490@163.com

1. The details for constant potential calculation of Ru_{S2}/V_{Bi}-BiTeBr.

The charged Ru_{S2}/V_{Bi}-BiTeBr slab, along with the compensating background charge, induces polarization of the electrolyte near the interface of metal and solution, generating an electrostatic potential profile that mimics the electric double layer. The electric potential of the Ru_{S2}/V_{Bi}-BiTeBr slab, referenced to the standard hydrogen electrode (SHE), is calculated as:

$$U_q(\mathrm{V/SHE}) = -4.6 - \varphi_q / e \,, \quad (1)$$

Here, -φ_q represents the work function of the charged Ru_{S2}/V_{Bi}-BiTeBr slab in aqueous media, while 4.6 V denotes the work function of the H_2/H^+ couple under standard conditions. The total energy of the charged system is subsequently corrected for the interaction with the background charge and the difference in the number of electrons in the system by:

$$E_{\mathrm{correction}} = \int_0^q \left\langle \overline{V_{\mathrm{tot}}} \right\rangle dQ + qU_q \,, \quad (2)$$

The total energy for the eleven charge values was then fitted to a quadratic function to yield the energy as a continuous function of potential. This quadratic form aligns with that of a capacitor formed by the charged slab and background charge system, expressed as follows:

$$E(U) = -1/2C(U\text{-}U_0)^2 + E_0 \,, \quad (3)$$

Where U_0 represents the potential of zero charge (PZC), E_0 denotes the energy at the PZC, and C stands for the surface capacitance. By employing the fitted quadratic functions for both the bare slab and slabs with H adsorption, the reaction energetics were computed as a function of electric potential.

The pH can alter the adsorption energies of reaction intermediates by modifying the electric potential. Consequently, adjusting the pH value leads to changes in electric potential on the SHE scale, thereby affecting the fixed potential on the reversible hydrogen electrode (RHE) scale according to the relationship:

$$U_{\mathrm{RHE}} = U_{\mathrm{SHE}} + k_b T \ln(10) pH / e \,, \quad (4)$$

In this work, the HER activities related to pH on the RHE scale were evaluated by computing reaction energies at the corresponding SHE potentials.

2. The discussion for the calculation results about different DFT methodologies.

We employed various DFT calculation methods to determine the ΔG_{H^*} values of the optimal catalyst (Ru_{S2}/V_{Bi}-BiTeBr) and analyze the influence of different methods on the outcomes. The results, listed in Table S1, demonstrate the influence of different methods on

the outcomes, with variations in ΔG_{H^*} values averaging approximately 0.13 eV, a relatively minor deviation. Previous studies extensively employed the PBE calculation method to investigate the HER and obtained results consistent with experiments [20,35,55–58]. Furthermore, the PBE method has a faster computational speed compared to the other two methods, balancing reasonable accuracy and lower computational costs for high-throughput calculations in our system. Therefore, using the PBE method in this work to study the catalytic activity of catalysts is feasible.

Figure S1. The phonon dispersion curves for the Ru_{S2}/V_{Bi}-BiTeBr.

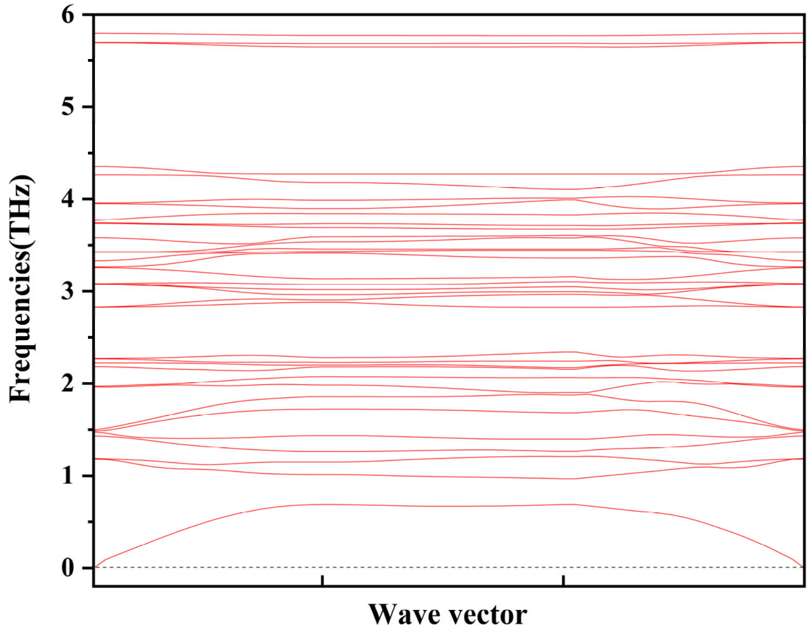

Table S1. The ΔG_{H^*} values of different DFT calculation methods for Ru_{S2}/V_{Bi}-BiTeBr.

Methods	ΔG_{H^*} (eV)
PBE	0.00
RPBE	0.14
revPBE	0.13

The initial coordinates used for DFT calculations:

```
BiTeBr_mp-33723_primitive
1.00000000000000
3.7497266585185787    -2.1649051859943307     0.0000000000000001
-0.0000000039213780     4.3298113587509546    -0.0000000000000001
0.0000000000000000    -0.0000000000000001     6.5616976639669646
Br    Te    Bi
1     1     1
Selective dynamics
Direct
0.6666669999999968   0.3333330000000032   0.7227678910503172
0.3333330000000032   0.6666669999999968   0.2705237604102706
0.0000000000000000   0.0000000000000000   0.0067083485394122
```
